# Network Changes in Insula and Amygdala Connectivity Accompany Implicit Suicidal Associations

**DOI:** 10.3389/fpsyt.2020.577628

**Published:** 2020-09-24

**Authors:** Elizabeth D. Ballard, Jessica R. Gilbert, Jessica S. Fields, Allison C. Nugent, Carlos A. Zarate

**Affiliations:** ^1^ Experimental Therapeutics and Pathophysiology Branch, National Institute of Mental Health, National Institutes of Health, Bethesda, MD, United States; ^2^ MEG Core Facility, National Institute of Mental Health, National Institutes of Health, Bethesda, MD, United States

**Keywords:** suicide, magnetoencephalography, suicide ideation, implicit association, dynamic causal modeling

## Abstract

Limited knowledge exists regarding the neurobiology of suicidal thoughts, given that there are currently no direct probes of the suicidal state. This pilot study used magnetoencephalography (MEG) to evaluate correlates of the implicit association between the self and death compared to the self and life as objective markers of suicide risk. Healthy volunteers (HVs; n=21) completed a modified version of the Suicide Implicit Association Task (S-IAT) during MEG scanning. Gamma power—which is considered a proxy measure of excitation-inhibition balance—was directly compared in the self-death/self-life contrast. As a proof-of-concept, the ability of dynamic causal modeling to categorize HVs versus four individuals with recent suicide crisis (SC) was evaluated. In HVs, enhanced gamma power in both amygdala and anterior insula were found for the self-death compared with self-life contrast. In addition, connectivity estimates between early visual cortex, anterior insula, and amygdala correctly categorized SC participants with 77% to 82% sensitivity and 80% to 85% specificity. These findings, which implicate network-level changes in salience network and amygdala connectivity in mediating suicidal associations, require further replication in larger samples. Direct probing of suicidal thoughts with the S-IAT may provide foundational markers of neural circuits associated with suicide risk.

## Introduction

Suicide remains critically understudied despite being a public health crisis. In the United States, suicide is the tenth leading cause of death, and 47,000 Americans died by suicide in 2017 alone (1). While increased efforts have attempted to address this crisis, the suicide rate has nevertheless risen by 30% over the past two decades (2). Despite its high prevalence, suicidal behavior remains difficult to predict. Direct screening of suicidal thoughts and behaviors is of limited value as individuals may minimize reporting of their suicidal thoughts due to concerns about hospitalization. Consequently, a critical need remains to identify objective markers of suicide risk and thus circumvent the limitations of self-report measures (3). In the absence of such markers, suicide risk will remain inadequately assessed and treated. In addition, understanding the neurobiology underlying suicidal thoughts could help develop targeted biological interventions (i.e., pharmacology, neurostimulation) that mitigate risk.

Although a number of neuroimaging studies have examined depression, bipolar disorder, or anxiety, the neuroimaging literature for suicide is relatively sparse. Structural and functional connectivity disturbances in ventral prefrontal cortex, insula, anterior cingulate, amygdala, hippocampus, and ventral striatum have all been implicated in suicidal thoughts and behaviors (4–7). Critically, most of these studies characterized suicide risk using lifetime history of suicide attempt—an event that could have occurred recently or years in the past. In contrast, very few studies have evaluated neural markers of suicide ideation in real time. In addition, neuroimaging analyses typically involve resting-state scans or tasks previously developed to assess correlates of mood disorders, including depression and bipolar disorder (e.g., evaluation of faces or decision-making), rather than tasks developed to directly assess suicide risk.

In tandem, these characteristics contribute to a comparative absence of neuroimaging paradigms and other biomarkers capable of directly probing the current suicidal state. Such paradigms could leverage new computational approaches—for example, by using advances in modeling network-level connectivity—to more precisely identify neural circuits associated with suicide risk and response to treatment. Such techniques (for instance, machine learning) have already been used in functional magnetic resonance imaging (fMRI) to classify suicidal individuals versus healthy controls using neural signatures for “life” and “death” words (8), setting the stage for innovative paradigms to more directly elucidate suicide risk.

One key evaluation in the clinical assessment of a suicidal individual is weighing wish to live versus wish to die, as the suicidal state often occurs when the wish to die overpowers the wish to live (9). The Suicide Implicit Association Task (S-IAT) is a behavioral marker for suicide risk that captures this distinction between the identification with life or death. Specifically, the S-IAT compares an individual’s implicit associations of self and life (the self-life condition) to self and death (the self-death condition); individuals with a history of suicide attempt and an increased risk of future suicide attempt have faster reaction times to the self-death condition (10). Studies have also found that S-IAT performance predicted past attempts and risk for future attempt above and beyond the use of other well-known risk factors; these findings have been replicated in emergency department, veteran, and internet samples (11–13).

A recent study from our laboratory adapted the S-IAT for fMRI in an effort to identify underlying neurobiological correlates associated with self-life and self-death implicit associations. In the pilot study of healthy volunteers (HVs), behavioral results obtained with the S-IAT were comparable to previously published results that administered the S-IAT outside of the scanner (14). In addition, the bilateral insula, middle occipital cortex, medial prefrontal cortex, and parahippocampal gyri were activated in the contrast between the self-death and self-life conditions. The activation of bilateral insula by the S-IAT is particularly notable given previous magnetoencephalography (MEG) findings from our group demonstrating a significant negative association between suicide ideation and gamma power in salience network regions, including the anterior insula, after ketamine administration (7). The results support the S-IAT as an ideal potential model to leverage computational approaches to studying suicide risk, given that it has been linked to prospective suicidal behavior, is feasible to administer in a neuroimaging environment, and may directly probe neural circuits related to suicide ideation.

The present MEG study used the S-IAT to examine the electrophysiological correlates of the implicit association of self with life and death. First, differences in gamma power were evaluated between self-life and self-death associations in a group of HVs in order to examine brain networks that mediate suicide-related thoughts. Gamma was selected because it is considered a putative surrogate marker of excitation-inhibition balance (15), has been suggested as a translational biomarker for psychiatric conditions such as depression, bipolar disorder (16), and schizophrenia (17), and has been previously associated with suicide ideation (7). Second, connectivity was modeled in a simple network activated by the task, which included early visual cortex, amygdala, and anterior insula. Connectivity was modeled using dynamic causal modeling (DCM), which fits a biologically plausible model of neural dynamics to measured electrophysiological signals. A Bayesian approach (18) was used to identify parameters important for task performance. Third, as a proof-of-concept, the clinical utility of this model was evaluated by examining the sensitivity and specificity of parameter estimates of connectivity in order to evaluate whether the task could identify a small group of individuals with a recent suicide crisis (SC) compared to HVs. The study sought to validate the S-IAT as a potential objective marker of suicide risk that would need to be evaluated in future prediction and treatment studies.

## Materials and Methods

### Participants

Twenty-one HVs and four participants who had recently experienced an SC—defined as suicide attempt or suicidal thoughts with intent to act within the previous two weeks—consented to participate in this study. HVs completed the study through a suicide-focused research protocol (NCT02543983) or a protocol studying functional and structural magnetic resonance imaging for mood and anxiety disorders (NCT00397111). All SC participants were recruited through the suicide-focused research protocol (NCT02543983); a description of the additional safety and recruiting efforts associated with this protocol have previously been published (19). All protocols were approved by the Combined Neuroscience Institutional Review Board at the National Institutes of Health in Bethesda, MD.

All participants were evaluated for psychiatric diagnoses using the Structured Clinical Interview for Axis I Diagnostic and Statistical Manual of Mental Disorders-IV (20). A history of Axis I DSM diagnoses, suicide attempts, or family history of psychiatric diagnoses or suicide attempt were exclusion factors for all HVs. SC participants were excluded if they had a diagnosis of schizophrenia or active substance dependence; all other psychiatric diagnoses and comorbidities were permitted. All participants had self-reported normal or corrected-to-normal vision.

### Suicide Implicit Association Task

Participants completed a modified version of the S-IAT (10) during MEG scanning. During the task, participants were instructed to categorize target words presented in the center of the screen (such as “die” or “live”) as belonging to the categories me, not me, life, and death (depicted on the upper right and left side of the screen) using a button box response (see **Supplemental Figure S1** for block design). The task was presented in an experimental run comprising eight blocks: four single category blocks (i.e., categorizing words as belonging to life versus death or me versus not me) and four “critical blocks”. During critical blocks, the categories of life/death were presented simultaneously with me/not me. Thus, in half of the critical blocks, participants categorized words as belonging to a pooled life/me or death/not me category or, conversely, categorized words as belonging to life/not me or death/me. Across blocks, screen side and the order of category pairings were randomized. Within blocks, target words were presented for 1.5 s, with an inter-trial fixation interval of 1.5 to 2.5 s (mean = 2 s). A total of 56 life/me and 56 death/me blocks were presented across the run. The adapted version of this task was previously validated for fMRI (14).

The primary behavioral outcome from the S-IAT is a *D-*score, which is the difference in mean reaction times between the self-death and self-life trials divided by the standard deviation of all critical trials. Positive D-scores represent a stronger association (i.e., faster reaction time) between the self and death, while negative D-scores represent a stronger association between the self and life. D-scores for HVs and SC participants were compared to zero using one-sample Wilcoxon signed-ranks tests to determine significance.

### MEG Acquisition and Preprocessing

Neuromagnetic data were collected using a 275-channel CTF system with SQUID-based axial gradiometers (VSM MedTech Ltd., Couquitlam, BC, Canada) housed in a magnetically-shielded room (Vacuumschmelze, Germany). Data were collected at 1200 Hz with a bandwidth of 0 to 300 Hz. Synthetic third order balancing was used for active noise cancellation. Offline, MEG data were first visually inspected and trials were removed where visible artifacts (e.g., head movements, jaw clenches, eye blinks, and muscle movements) were present. In addition, individual channels showing excessive sensor noise were marked as bad and removed from the analysis. Data were then bandpass-filtered from 1 to 58 Hz and epoched from −100 to 1000 ms peristimulus time. The analysis routines available in the academic freeware SPM12 (Wellcome Trust Centre for Neuroimaging, London, UK, http://www.fil.ion.ucl.ac.uk/spm/) were used for data processing.

### Source Localization and Source Activity Extraction

The multiple sparse priors routine implemented in SPM12 (http://www.fil.ion.ucl.ac.uk/spm/) was used to identify gamma frequency (30–58 Hz) sources of activity from each participant’s sensor-level data over a peristimulus event time window from −100 to 500 ms. Induced responses to reading target words were localized to 512 potential mesh points using a variational Bayesian approach following coregistration of sensor positions to a canonical template brain. Participant-level activation maps were constructed following inversion of the data separately for all participants, without prior constraints on source locations. Following this inversion, statistical maps of group activity were computed, and a mixed-effects ANOVA was used to define source-localized cortical regions showing a main effect of the task, thresholded at p<0.05 family-wise error (FWE) correction. In addition, self-death to self-life trials were directly contrasted using a more liberal criterion of p<0.05, uncorrected, to generate additional candidate regions of interest (ROIs) for the DCM analysis.

### Dynamic Causal Modeling

**Figure 1:**
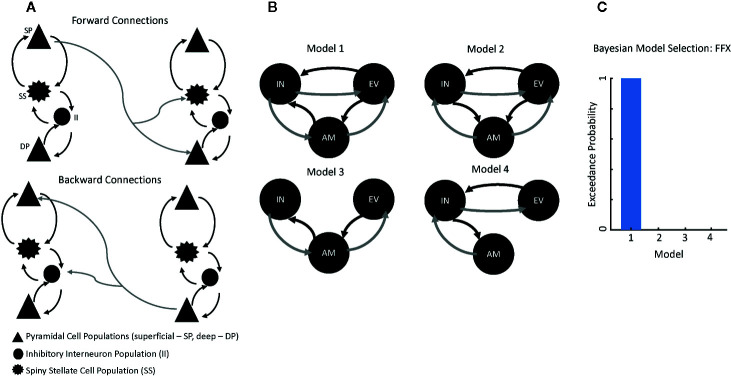
Modeling Connectivity for the Suicide Implicit Association Task (S-IAT). **(A)** Conductance-based neural mass model for dynamic causal modeling (DCM) for electrophysiology, which includes excitatory and inhibitory connection parameters from four distinct cell layers: superficial pyramidal cells (SPs), spiny stellate cells (SSs), deep pyramidal cells (DPs), and inhibitory interneurons (IIs). Forward connections originate from SPs to both SSs and DPs. Backward connections originate from DPs to both SPs and IIs. **(B)** Four plausible models were constructed to account for message-passing between early visual cortex, amygdala, and insula. **(C)** Results from Bayesian model selection to adjudicate between the four models; the strongest evidence was found for Model 1, which had fully reciprocated forward and backward connections between each region of interest.

DCM uses a biophysical model of neural responses based on neural mass models to predict recorded electrophysiological data (21). The present study specifically used a conductance-based neural mass model for DCM for electrophysiology—the canonical microcircuit (“CMC”) model as implemented in SPM12—to model responses between our ROIs. The CMC includes excitatory and inhibitory connection parameters from four distinct cell layers: superficial pyramidal cells, spiny stellate cells, deep pyramidal cells, and inhibitory interneurons (**Figure 1A**). Within the model, superficial pyramidal cells encode and carry feedforward signaling to spiny stellate cells and deep pyramidal cells, while deep pyramidal cells carry feedback signaling to both superficial pyramidal cells and inhibitory interneurons (**Figure 1A**). More detailed information on the model architecture can be found in the spm_fx_cmc.m file, freely available in SPM12. Thalamic (stimulus-bound) input was modeled with a Gaussian bump function that drove activity in left early visual cortex in our analysis.

Because our aim was to characterize differences in connectivity as a function of trial type (i.e., self-life, self-death), we focused on three regions in order to model forward and backward connections in our network: left early visual cortex (−2, −92, −4), left amygdala (−20, −10, −30), and left insula (−50, 10, 2). Four plausible models were constructed to account for message-passing between these regions (**Figure 1B**) in our HVs only. In Model 1, forward connections carried signals from early visual cortex to both amygdala and insula as well as from amygdala to insula. Recurrent backward connections ensured reciprocal connectivity between these regions in the model and all subsequent models. Model 2 included forward connections from early visual cortex to both amygdala and insula as well as from insula to amygdala. Model 3 included forward connections from early visual cortex to amygdala and from amygdala to insula. Finally, Model 4 included forward connections from early visual cortex to insula and from insula to amygdala.

All models included trial-specific (i.e., self-life versus self-death) modulations on all connections. To adjudicate between these models, the negative free energy bound on the log-model evidence was used; the model with the highest log-model evidence was selected for subsequent analyses. This model architecture was then fitted to the SC participants. Parameter estimates from the model were extracted for all participants to compare intrinsic, region-to-region connectivity and trial-specific (i.e., self-life, self-death) modulations on these connections.

For the DCM analyses, MEG activity for the extracted time series was fitted over 1 to 500 ms peristimulus time in a wide frequency band from 1 to 58 Hz to capture cross-spectral density estimates from each ROI. For computational efficiency, DCM optimizes a posterior density over free parameters (parameterized by its mean and covariance) *via* a standard variational Bayesian inversion procedure (22). Model inversion results in optimized parameters of different receptor-mediated synaptic responses given the model architecture that best predict the given data. This study was specifically interested in intrinsic connectivity estimates and trial effects (i.e., self-life versus self-death) of the optimized parameters.

To determine the mixture of intrinsic, region-to-region connectivity parameters and their modulation by task (i.e., self-life compared with self-death trials during critical blocks) mediating both the group effect (mean across all participants) and the difference between groups (SC versus HV), a second-level modeling extension of DCM called parametric empirical Bayesian analysis (18) was applied. This analysis refits a full model where all parameters can covary according to grouping (here, all participants). It also provides reduced models where smaller combinations of parameters are considered and informed by differences between groups. We specifically assessed the group effect on all region-to-region connections and on modulatory connections in our second-level design matrix, where the first column represented the average effect for the entire group (i.e., ones for all participants) while the second column tested the difference between groups (i.e., zeros for HVs, ones for SC participants).

### Exploratory Dynamic Causal Modeling Analysis

As an additional exploratory analysis, we specifically harvested the parameter estimates identified as having a “significant” group difference (i.e., HVs versus SCs) using a posterior probability of greater than 0.95 and determined whether these parameter estimates could be used to classify SC participants versus HVs. Here, a control average Gaussian probability density function was constructed using the overall HV mean and variability as the mean and variance of the control group. For the SC participants, average SC mean and variability were used as the mean and variance of the SC group. To calculate specificity for HVs, a leave-one-out cross-validation method was used where a control parameter was removed from the average density and compared to the average density from the remaining participants using a threshold of P>0.95 probable difference level. To calculate sensitivity in the SC group, the average control probability density was compared to the conditional density from each SC participant separately. Non-overlapping probability density was used to quantify the difference in parameter estimates.

## Results

The 21 HVs had a mean age of 27.7 (standard deviation (SD)=4.9) years and included 15 females. The four SC participants had a mean age of 37.8 (SD=11.0) years and included two females. SC participants had a mean of 3.3 past suicide attempts (SD=4.5) and 3.5 (SD=2.7) Axis I psychiatric diagnoses. SC participants were moderately to severely depressed (mean Montgomery-Asberg Depression Rating Scale (MADRS)=28.0, SD=7.0) and reported significant hopelessness (mean Beck Hopelessness Scale=17.5, SD=2.1) with moderate to severe lifetime suicidal thoughts (mean Scale for Suicide Ideation Worst Episode=29.5, SD=5.8).

Behaviorally on the S-IAT, HV scores ranged from −1.30 to 0.55, with five scores over 0 (5/21 or 24%, representing a slight death-me bias). SC participant scores ranged from −0.5 to .27, with two scores (2/4 or 50%) representing a slight death-me bias. When comparing each group to zero, HVs showed a significant self-life bias (Z = −2.76, p<0.01, mean = −0.297, SD = 0.44) while SC participants showed no bias towards either self-life or self-death (p = 0.88, mean = −0.083, SD=0.32).

**Figure 2:**
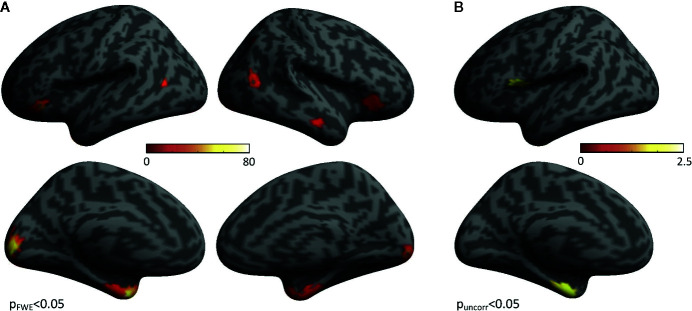
Regions Activated During the Task. **(A)** Group-level statistical activation maps of stimulus-induced gamma-band activity from Suicide Implicit Association Task (S-IAT) administration. **(B)** Group-level statistical activation maps of stimulus-induced gamma-band activity from the contrast between self-death and self-life trials as part of S-IAT administration.

In HVs, group-level statistical activation maps demonstrated stimulus-induced gamma-band activity in a network of brain regions including bilateral early visual cortex, bilateral occipito-temporal cortex, bilateral anterior ventral temporal cortex, and bilateral inferior frontal cortex (**Figure 2A**). In addition, when directly contrasting self-life and self-death trials, increased gamma power for self-death trials compared with self-life trials was found in the bilateral anterior ventral temporal cortex, encompassing parahippocampal cortex and amygdala in addition to left insular cortex (**Figure 2B**). No regions demonstrated enhanced gamma power for self-life compared with self-death trials.

Four plausible models for message-passing between early visual cortex, amygdala, and insula were constructed using DCM (**Figure 1B**). Using Bayesian model selection to adjudicate between these models, the strongest evidence was found for Model 1, which had fully reciprocated forward and backward connections between each ROI (**Figure 1C**). Model fits were then computed for Model 1 by correlating the estimated model spectra to the data spectra within each ROI. Model fits were then compared between HV and SC participants to confirm there was no biasing of fits for SC participants. Model 1 consistently recapitulated spectral data across groups (HV mean=0.709, HV SE=0.050; SC mean=0.681, SC SE=0.185).

**Figure 3:**
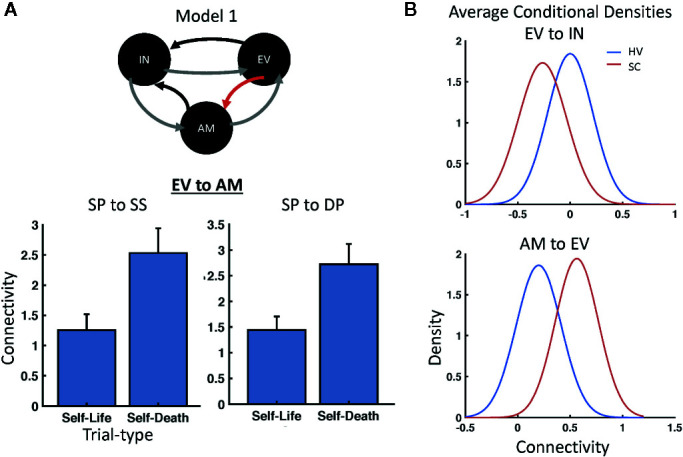
Modulatory and Group Effects. **(A)** Parametric Empirical Bayesian (PEB) analysis identified modulations on the forward connections from early visual cortex (EV) to amygdala (AM) based on trial type; increased connectivity was found for self-death compared with self-life trials across participants for both superficial pyramidal (SP) to spiny stellate (SS) cell and SP to deep pyramidal (DP) cell connections. **(B)** PEB also identified significant group differences in connectivity between healthy volunteer (HV) and suicide crisis (SC) participants for both the forward connection between EV and insula (IN) carried *via* superficial pyramidal cells to SS cells and the backward connection from AM to EV carried by DP to SP cells.

Parametric empirical Bayes was used to test for random effects of model parameters, testing both the average effect across participants (i.e., identifying parameters important for the task across participants) as well as the difference between HVs and SCs directly. The analysis focused on parameters that exhibited significant effects by focusing on those parameters having a posterior probability of P>0.95 (see **Table 1** for a full list of parameters). Significant parameters mediating the average effect across participants included the forward connection carried by superficial pyramidal cells to spiny stellate cells between early visual cortex and amygdala, in addition to the forward connection carried by superficial pyramidal cells to deep pyramidal cells between those same regions. In addition, significant task modulations (i.e., self-death versus self-life) were found on the forward connections between early visual cortex and amygdala. For both forward connections between early visual cortex and amygdala (i.e., superficial pyramidal cell to spiny stellate cell, superficial pyramidal cell to deep pyramidal cell), self-death trials increased the estimated connectivity compared with self-life trials (**Figure 3A**). Significant group differences (i.e., HV versus SC participants) were found for both: 1) the forward connection between early visual cortex and insula carried *via* superficial pyramidal cells to spiny stellate cells, and 2) the backward connection from amygdala to early visual cortex carried by deep pyramidal cells to superficial pyramidal cells. The forward connection demonstrated reduced connectivity estimates for SC participants relative to HVs, while the backward connection demonstrated enhanced connectivity estimates for SC participants relative to HVs (**Figure 3B**).

**Table 1:** Parameters mediating the average effect and difference between groups.

	Parameter	Parameter Estimate (Ep)	Posterior Probability (Pp)
**Average Effects**			
1	Forward Connection (SP to SS)—EV to AM	−0.3035	1
2	Forward Connection (SP to DP)—EV to AM	−0.2287	1
3	Trial Modulation – Forward Connections—EV to AM	−0.4451	1
4	Backward Connection (DP to SP)—IN to EV	0.144	0.6059
5	Backward Connection (DP to SP)—IN to AM	0.0695	0.505
6	Forward Connection (SP to SS)—EV to IN	−0.0696	0.503
7	Forward Connection (SP to SS)—AM to IN	−0.0833	0.502
**Group Differences**			
1	Forward Connection (SP to SS)—EV to IN	−0.5904	1
2	Backward Connection (DP to SP)—AM to EV	0.5477	1
3	Backward Connection (DP to II)—IN to EV	0.247	0.54
4	Backward Connection (DP to II)—AM to EV	0.2341	0.528
5	Backward Connection (DP to SP)—IN to EV	0.1824	0.521
6	Backward Connection (DP to SP)—IN to AM	−0.1708	0.504

Parametric empirical Bayes was used to identify the mixing of parameters that contributed to both the average effect across participants and the difference between groups (healthy volunteers (HVs) versus participants experiencing a suicide crisis (SC)). Meaningful parameters were defined as those with a posterior probability (Pp) of greater than 0.95. Significant effects across participants were found on the forward connections between early visual cortex (EV) and amygdala (AM), carried by superficial pyramidal cells (SPs) to both spiny stellate cells (SSs) and deep pyramidal cells (DPs). Significant trial modulations (i.e., self-death versus self-life) across participants were also found on the forward connections from EV to AM. Significant group differences were found on the forward connections from EV to insula (IN) carried by SP to SS cells, and on the backward connections from AM to EV carried by DP to SP cells. II=inhibitory interneurons.

Sensitivity and specificity were subsequently calculated for both the forward connection from early visual cortex to insula and the backward connection from amygdala to early visual cortex. The average conditional density plots for each parameter are illustrated in **Figure 3B**. Sensitivity estimates to identify SC participants compared to HVs for the forward connection carried by superficial pyramidal cells to spiny stellate cells between early visual cortex and insula were 50%, 74%, 89%, and 93% (mean=77%). Sensitivity estimates for the backward connection carried by deep pyramidal cells to superficial pyramidal cells between amygdala and early visual cortex were 59%, 73%, 96%, and 100% (mean=82%). Using a threshold of P>0.95 probable difference level to determine specificity within our HVs, a true negative rate of 85% was found for the forward connection for early visual cortex to insula and 80% for the backward connection from amygdala to early visual cortex.

## Discussion

This study is the first to use MEG to evaluate potential electrophysiological correlates of the implicit association between the self and death compared to the self and life as a potential objective marker of suicide risk. In the present study, increases in gamma were found in a network of regions including bilateral early visual cortex, anterior ventral temporal cortex (including parahippocampal cortex and amygdala), and inferior frontal gyrus. In addition, when comparing self-death/self-life associations directly, increased gamma power was found in amygdala and anterior insula for self-death categorizations compared with self-life categorizations. These findings echo previous fMRI findings (14) that demonstrated similar patterns of S-IAT behavioral responses in HVs as well as enhanced BOLD activation in the anterior insula, middle occipital cortex, medial prefrontal cortex, and parahippocampal gyri for self-death compared to self-life categorizations.

As a proof-of-concept, the ability of MEG estimates of connectivity during the S-IAT to characterize HVs compared to a small sample of participants who had recently experienced an SC was evaluated using DCM. SC participants were categorized with 77% sensitivity and 85% specificity using the forward connectivity from early visual cortex to insula and with 82% sensitivity and 80% specificity using the backward connectivity from amygdala to early visual cortex. These findings suggest that electrophysiological estimates of connectivity during S-IAT administration may be a useful objective marker of suicide risk and further implicate the salience network and amygdala in mediating suicidal implicit associations.

Suicide research may be particularly well-suited for computational modeling approaches seeking to identify underlying neural, cellular, and molecular systems associated with the decision to take one’s life. Similar approaches in the depression literature identified fundamental processes associated with depression and anhedonia, such as reward prediction error, reward sensitivity, or learning (23); parallel approaches in suicide could consider fundamental suicidal processes such as thinking about death (8), associating the self with death, or the decision to act on suicidal thoughts. Such an approach could more precisely identify intermediate phenotypes as well as treatment targets for suicidal behavior. For example, activity in the insula and salience network have been implicated in models of psychopathology (24), may predict response to psychiatric medications as compared to psychotherapy (25), and may mediate changes in default mode connectivity in response to ketamine, an intervention with known suicide ideation effects (26, 27). Thus, tasks that modulate the insula could be fundamental for developing potential biomarkers of response in individuals at risk for suicide.

Limitations of the study include the small sample size, as only four recently suicidal participants were included to evaluate the feasibility of this type of modeling. Future studies with larger samples are needed to evaluate the effects of current suicidal thoughts, past suicide attempt, and severity of depressive symptoms on response to this task. Because DCM is model-dependent, four biologically plausible models of message-passing were constructed using three ROIs; however, further modeling work should more thoroughly evaluate regions that mediate suicidal thoughts. Lastly, several versions of the S-IAT exist, including some whose tasks involve suicide method or non-suicidal self-harm; thus, another version of the task may more precisely target implicit suicide associations (11).

Nevertheless, these data provide valuable foundational knowledge about the electrophysiological correlates of implicit associations of suicidal thoughts. These preliminary results highlight the feasibility and potential utility of suicide-specific probes of neural circuits that can be used in suicide prediction as well as in the evaluation of treatment response in larger, more comprehensive samples.

## Data Availability Statement

The raw data supporting the conclusions of this article will be made available by the authors, without undue reservation.

## Ethics Statement

The studies involving human participants were reviewed and approved by Combined Neuroscience Institutional Review Board at the National Institutes of Health in Bethesda, MD, USA. The patients/participants provided their written informed consent to participate in this study.

## Author Contributions

EB: conceptualized the study; designed the study; drafted/revised the manuscript; approved the final version of the manuscript. JG: designed the study; drafted/revised the manuscript; conducted the statistical analysis; approved the final version of the manuscript. JF: conducted the literature search; edited the manuscript for critical intellectual content; assisted in statistical design, analysis, and interpretation; approved the final version of the manuscript. AN: edited the manuscript for critical intellectual content; provided research supervision; approved the final version of the manuscript. CZ: edited the manuscript for critical intellectual content; provided research supervision; approved the final version of the manuscript.

## Funding

Funding for this work was supported by the Intramural Research Program at the National Institute of Mental Health, National Institutes of Health (IRP-NIMH-NIH; ZIAMH002857; NCT02543983 and NCT00397111); by a NARSAD Independent Investigator Award to CZ; and by a Brain and Behavior Mood Disorders Research Award to CZ. These organizations had no further role in study design; in the collection, analysis, or interpretation of data; in the writing of the report; or in the decision to submit the paper for publication.

## Conflict of Interest

CZ is listed as a co-inventor on a patent for the use of ketamine in major depression and suicidal ideation; as a co-inventor on a patent for the use of (2*R*,6*R*)-hydroxynorketamine, (*S*)-dehydronorketamine, and other stereoisomeric dehydro and hydroxylated metabolites of (*R,S*)-ketamine metabolites in the treatment of depression and neuropathic pain; and as a co-inventor on a patent application for the use of (2*R*,6*R*)-hydroxynorketamine and (2*S*,6*S*)-hydroxynorketamine in the treatment of depression, anxiety, anhedonia, suicidal ideation, and post-traumatic stress disorders. He has assigned his patent rights to the U.S. government but will share a percentage of any royalties that may be received by the government.

The remaining authors declare that the research was conducted in the absence of any commercial or financial relationships that could be construed as a potential conflict of interest.

## Supplementary Material

Supplementary Figure 1 Task Design for Suicide Implicit Association Task (S-IAT) Administration. This visual depiction shows the task design for the S-IAT task, including the “critical blocks” (denoted in bold) in which words are categorized into both “death/life” and “me/not me” categories. Trial order was counterbalanced across participants so that half the participants were presented “Part 1” followed by a 10 s rest period and then “Part 2”. The other half of the participants were presented “Part 2” followed by a 10-s rest period and then “Part 1”.
