# Supplementary figures and images for: Network Changes in Insula and Amygdala Connectivity Accompany Implicit Suicidal Associations
Source: Front Psychiatry. 2020 Sep 24;11:577628. doi: 10.3389/fpsyt.2020.577628 (PMC7543650; doi:10.3389/fpsyt.2020.577628)

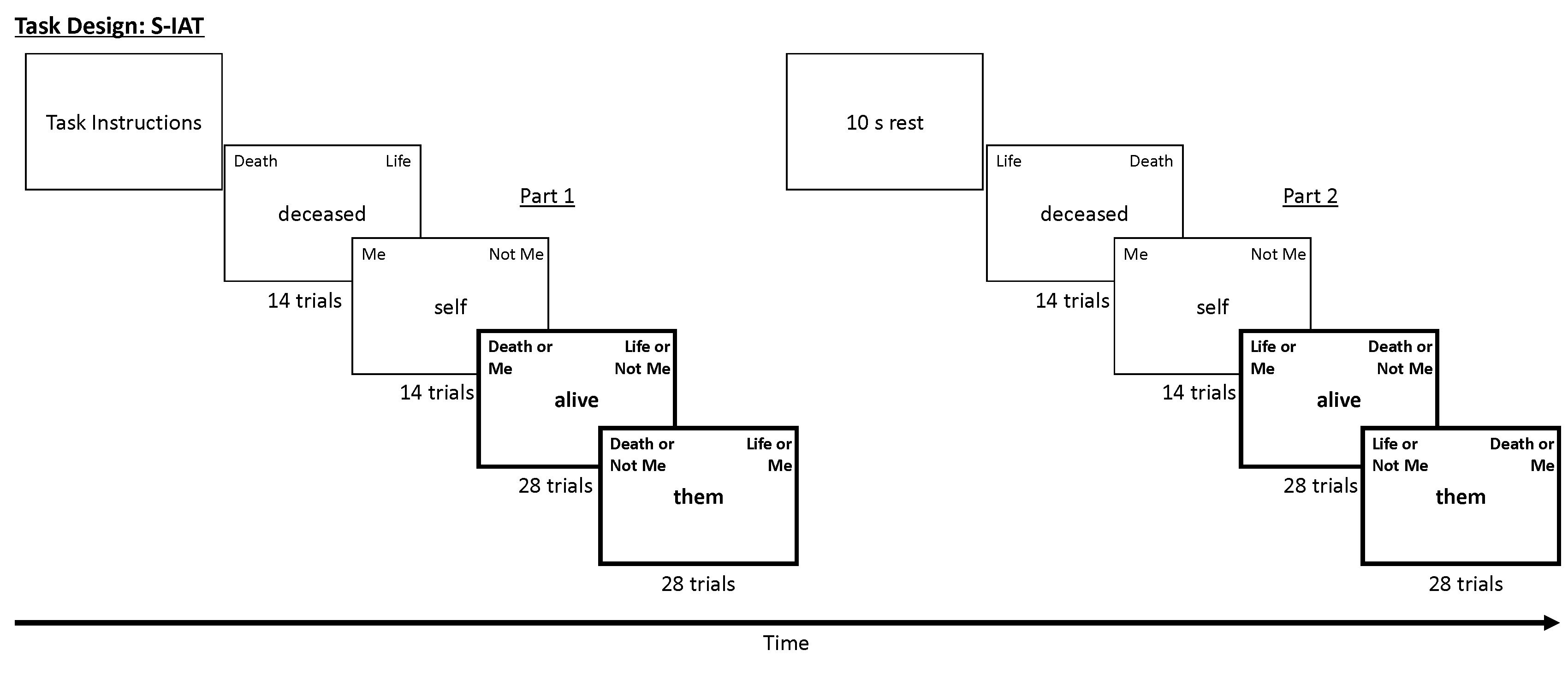

Supplement: Supplementary Figure 1 — Task Design for Suicide Implicit Association Task (S-IAT) Administration. This visual depiction shows the task design for the S-IAT task, including the “critical blocks” (denoted in bold) in which words are categorized into both “death/life” and “me/not me” categories. Trial order was counterbalanced across participants so that half the participants were presented “Part 1” followed by a 10 s rest period and then “Part 2”. The other half of the participants were presented “Part 2” followed by a 10-s rest period and then “Part 1”. [file Image_1.jpeg]
